# Improved Field-Based Soybean Seed Counting and Localization with Feature Level Considered

**DOI:** 10.34133/plantphenomics.0026

**Published:** 2023-03-15

**Authors:** Jiangsan Zhao, Akito Kaga, Tetsuya Yamada, Kunihiko Komatsu, Kaori Hirata, Akio Kikuchi, Masayuki Hirafuji, Seishi Ninomiya, Wei Guo

**Affiliations:** ^1^ Graduate School of Agriculture and Life Sciences, The University of Tokyo, Tokyo, Japan.; ^2^ Institute of Crop Sciences, National Agriculture and Food Research Organization, Tsukuba, Ibaraki, Japan.; ^3^ Western Region Agricultural Research Center, National Agriculture and Food Research Organization, Fukuyama, Hiroshima, Japan.; ^4^ Tohoku Agricultural Research Center, National Agriculture and Food Research Organization, Morioka, Iwate, Japan.

## Abstract

Developing automated soybean seed counting tools will help automate yield prediction before harvesting and improving selection efficiency in breeding programs. An integrated approach for counting and localization is ideal for subsequent analysis. The traditional method of object counting is labor-intensive and error-prone and has low localization accuracy. To quantify soybean seed directly rather than sequentially, we propose a P2PNet-Soy method. Several strategies were considered to adjust the architecture and subsequent postprocessing to maximize model performance in seed counting and localization. First, unsupervised clustering was applied to merge closely located overcounts. Second, low-level features were included with high-level features to provide more information. Third, atrous convolution with different kernel sizes was applied to low- and high-level features to extract scale-invariant features to factor in soybean size variation. Fourth, channel and spatial attention effectively separated the foreground and background for easier soybean seed counting and localization. At last, the input image was added to these extracted features to improve model performance. Using 24 soybean accessions as experimental materials, we trained the model on field images of individual soybean plants obtained from one side and tested them on images obtained from the opposite side, with all the above strategies. The superiority of the proposed P2PNet-Soy in soybean seed counting and localization over the original P2PNet was confirmed by a reduction in the value of the mean absolute error, from 105.55 to 12.94. Furthermore, the trained model worked effectively on images obtained directly from the field without background interference.

## Introduction

Soybean is an important protein source for animals and humans worldwide [1]. Therefore, achieving high crop yields is a common criterion and ultimate goal in most breeding programs, whereby considerable efforts are invested in evaluating yield during soybean breeding [2–4]. However, grain yield is usually measured only after harvest using labor-intensive methods [5]. Thus, an effective yield prediction method would greatly help breeders to focus on solving biological problems rather than being distracted by troublesome yield prediction models [6–9]. Simultaneously, the distribution of soybean seed setting position is essential for breeding, because seeds set in the lower plant segment can be lost during harvesting [10].

Several studies indicate that soybean yield strongly correlates with the number of pods per plant. Indirect yield prediction models using plant phenotypic or chemical traits generally show low performance [4]. However, the number of seeds varies among pods on individual plants, which is particularly important when different soybean cultivars are compared for breeding purposes [11]. Knowledge of the number of seeds and location of the plants helps to track the yield potential of a cultivar and make wiser on-farm decisions throughout plant reproductive growth. Intuitively, seed counting should be more directly related to yield than pod counting, potentially improving soybean yield prediction accuracy in practice. However, accurate soybean seed counting is not a trivial matter; manual seed counting is labor-intensive, time-consuming, and error-prone, especially after a long period of hard work, particularly when performed in the field [4,12]. Pod counting was selected for yield prediction because pods are larger than seeds and, thus, assumed to be easier to manage than seed counting. Coincidently, Wei and Molin [4] also found a strong correlation between soybean pod number and yield. Similarly, using deep learning, Riera et al. [9] deployed detection-based soybean pod counting. Although multiview images were used for pod detection, they were low in performance and lacked future application potential. Alternatively, there are also seed-counting-based multiview images; however, studies on seed counting are restricted to strictly controlled laboratory conditions. One such study showed an acceptable estimation accuracy of the deep-learning-based method in counting the number of seeds per soybean pod [13]. In another study, the total number of seeds of the collected pods within an image was predicted with high accuracy [12]. However, in all these cases under controlled conditions, seed counting was largely successful because of the prevalent homogeneous background. Under such conditions, pods were manually placed without overlapping, and pseudo-bounding boxes generated on the basis of point annotations were used to assist seed localization, making the task relatively easy to manage. However, these highly sophisticated methods developed in the laboratory would surely fail in the field because the background, natural daylight conditions, and the level of pod overlap differ greatly in field images. Therefore, more advanced methods that can deal with seed counting under difficult conditions, such as complex backgrounds, extensively overlapping pods, and varying light conditions, are urgently needed. Moreover, advanced methods that can count and locate soybean seeds simultaneously are preferable when easily acquired field true-color red, green, and blue color images of soybean plants can be used, especially in a high-throughput manner. Furthermore, automated seed counting is preferred for improving breeding efficiency.

Manual seed counting is labor-intensive, costly, time-consuming, and error-prone [14,15]. Traditional image-based counting methods require images to be relatively uniform and of high quality, but, in general, their level of precision is rather poor [16–19], usually because color and/or texture information is effectively captured by old-fashioned algorithms in computer vision, especially when there are large variations in counting targets and background information [20].

Deep learning has evolved with respect to image processing, thereby making image-based object counting much easier than before [21–23]. Deep-learning-based automatic soybean seed counting can be a highly valuable tool to speed up yield prediction and subsequent breeding processes, especially considering its ability to overcome almost all shortcomings of manual and traditional image-based seed counting. The common deep-learning-based method for object counting is a pipeline for detecting objects first, followed by counting the predicted bounding boxes [21,24]. However, this comes at the cost of manual and expensive bounding box annotations. Moreover, overlapping bounding boxes are difficult to suppress when targets are located too close to each other [25]. Furthermore, object detection and image segmentation should not be prerequisites for counting, as both are generally considered much more challenging tasks than counting in computer vision. The popularity of detection-based counting methods might be due to the well-researched and off-the-shelf frameworks readily available for use, such as faster R-CNN [21], YOLO series [22,26,27], and other fully convolutional networks [28], UNET [23], and DeepLab [29].

Instead of a sequential approach, counting should be addressed more directly using cheap dotted annotations. Direct counting by regression is another category of deep learning for object counting that involves connecting the counts to the target image with the assistance of dot labels [30,31]. However, this technique does not provide the exact locations of the counted objects, making it a black box in terms of what has been counted and the locations of the counted objects in the image. The TasselNet series [32–34] focuses on accurately counting the number of tassels using regression-based methods. However, the visualized density maps of the predicted objects could not be explained by intuition.

The recently proposed P2PNet [35] method makes point counting much easier. In addition, the underlying basic theory is relatively straightforward, locating and counting these points by directly training a model with points as labels, rather than traditional bounding boxes or density maps created from dotted labels. However, P2PNet showed low performance when used directly for soybean seed counting, which can be explained by several reasons. First, the original P2PNet was developed to count all objects of the same category but with no disturbance from objects similar to the background. In contrast, field soybean counting becomes extremely difficult when other seed-bearing soybean plants appear beside and behind the target plants. Thus, the background of field soybean plant images is complex, and, unavoidably, seeds from neighboring plants often appear in the background, thus reducing model performance. Second, substantial overpredictions regarding the corresponding true seed locations were observed. Hence, more effective methods for merging overcounts to improve seed localization and counting accuracy should be included. Third, different levels of features were merged through a simple upsampling process, although the advantages of different levels of features have not been fully explored. High-level features contain global-context-aware information, which is more suitable for locating the salient regions. In contrast, low-level features maintain more spatial structural details, which are more sensitive in detecting object boundaries [36]. Only high-level features were used in the original model, which may have lowered its effectiveness. Finally, the scale of the objects was not considered. In crowd counting, the size of the targets is relatively homogeneous, whereas the size of soybean seeds is more diverse in breeding materials. Moreover, the heavily overlapped seeds also affect the visible size of the counted seeds heavily which can be used for feature extraction. Therefore, in this study, we attempted to upgrade the original P2PNet to P2PNet-Soy, which is a fully capable soybean seed counting method for field soybean images by solving the problems faced by directly transferred P2PNet.

## Materials and Methods

This section describes data collection and preprocessing, which constitutes the framework for soybean seed counting and localization.

### Field conditions and experimental design

Six hundred and fifty soybean accessions were planted in 3-row plots in the field at National Agriculture and Food Research Organization, Tsukuba, Ibaraki, Japan (36°01′25.6″ N, 140°06′59.1″ E). Five plants per accession were grown with an inter-row spacing of 0.8 m and a hill spacing of 0.2 m. Seeds were sown on 2021 June 10. A starter fertilizer containing N (3 g/m^2^), P_2_O_5_ (10 g/m^2^), and K_2_O (10 g/m^2^) was applied. Intertillage was conducted 1 month after sowing. Insecticides and fungicides were sprayed at 2-week intervals from July to September. A total of 24 accessions showing similar maturity were selected in this study. Five plants per accession in the same row were imaged, and the total number of seeds was counted.

### Field data acquisition and preprocessing

**Fig. 1.**
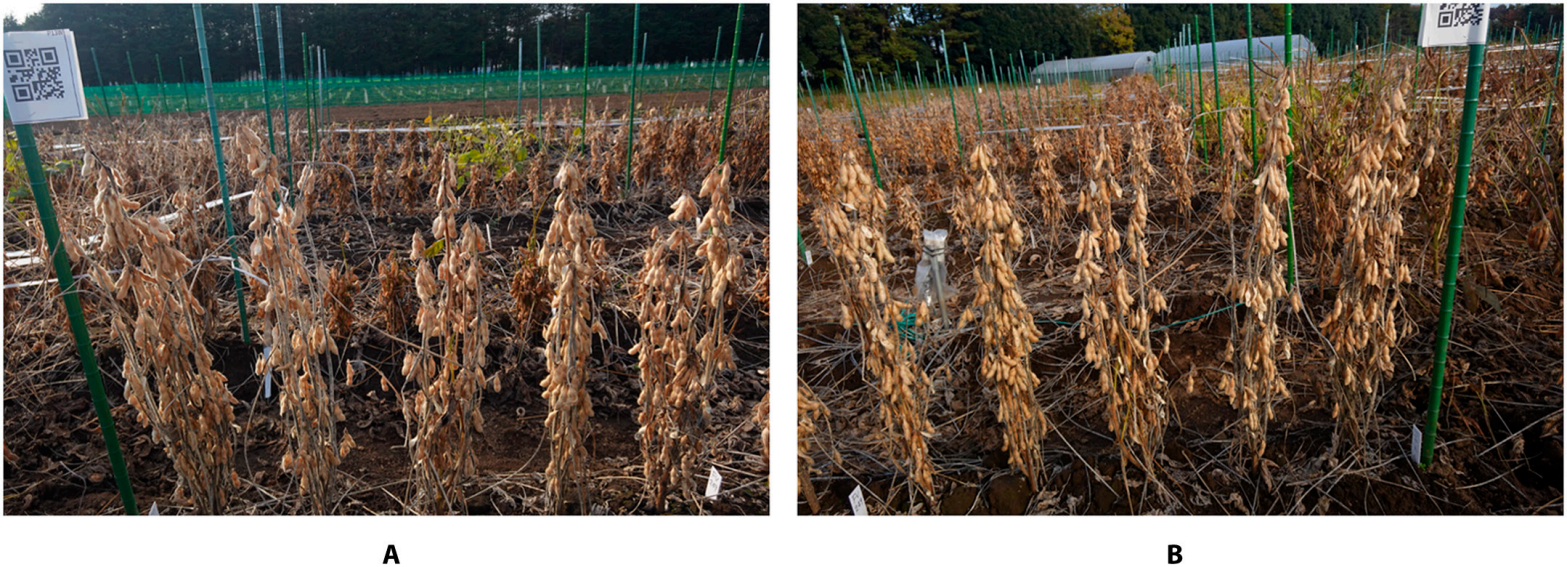
Field images of soybean plants taken by a hand-held camera. (A) Image of the plants from one side. (B) Image of the same plants from the opposite side.

**Fig. 2.**
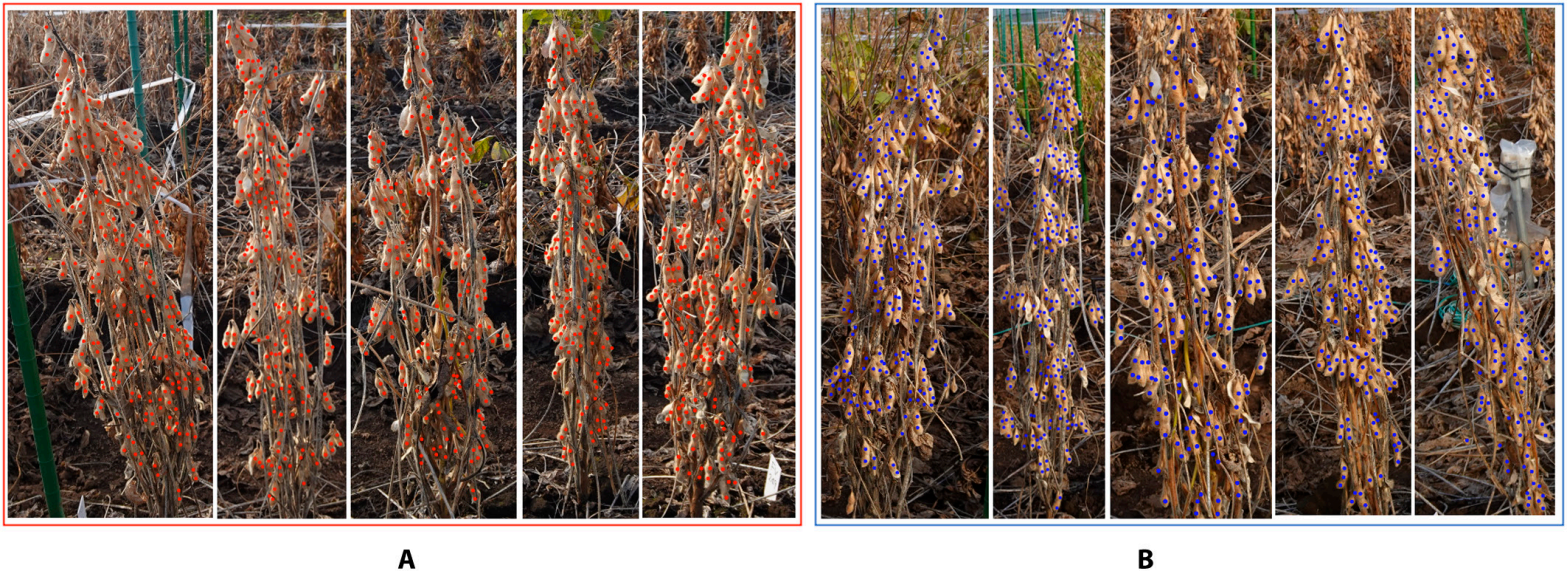
Dot-annotated cropped individual soybean plants. (A) Annotated individual plants from one side. (B) Annotated individual plants from the opposite side.

Both sides of the 5 plants per accession were imaged using a hand-held camera (DSC-RX0M2, Sony, Japan) to reduce hidden or overlapping effects from the densely located soybean seeds (Fig. 1). To prepare the training and testing datasets, we cropped the plants individually from the block images (Fig. 2). Because of the small distance between neighboring plants, partial inclusion of segments from neighboring plants was unavoidable in individual plant images. The seeds of each plant were dot-annotated (Fig. 2) by experienced technicians from the Field Phenomics Lab at the University of Tokyo. Only those seeds belonging to the target cropped individual plant were annotated, while seeds from the neighboring plants from both sides and background were ignored. A seed was annotated as long as 1/10 of its full size was visible in the image to ensure that the number of annotated seeds was as close as possible to the number of seeds counted by destructive sampling (Fig. 2). A total of 374 images of individual plants were prepared as the experimental dataset for this study; the rest of them were not included because of pod shattering.

### Deep learning framework for soybean seed counting and localization

The original P2PNet is a purely point-based framework [37]. It directly takes point annotations as masks and can thus provide the exact location of the predicted targets rather than a pure number or density map of objects. The model is trained to predict points as close to the ground-truth points as possible. The final prediction of P2PNet is based on the summation of 2 branches: regression, which indicates the locations of the points, and classification, which is based on the confidence scores of the predicted points. Both branches share higher-level features extracted by VGG-16 [38]. In this study, high-level and low-level features were defined as conv3-3, conv4-3, and conv5-3 layers, as well as another group consisting of conv1-3 and conv2-3 layers of VGG-16. The network is not only structurally clean, intuitive, and straightforward but also determines both the number and locations of counted objects simultaneously. Therefore, it was taken as the basis for managing soybean seed counting tasks.

**Fig. 3.**
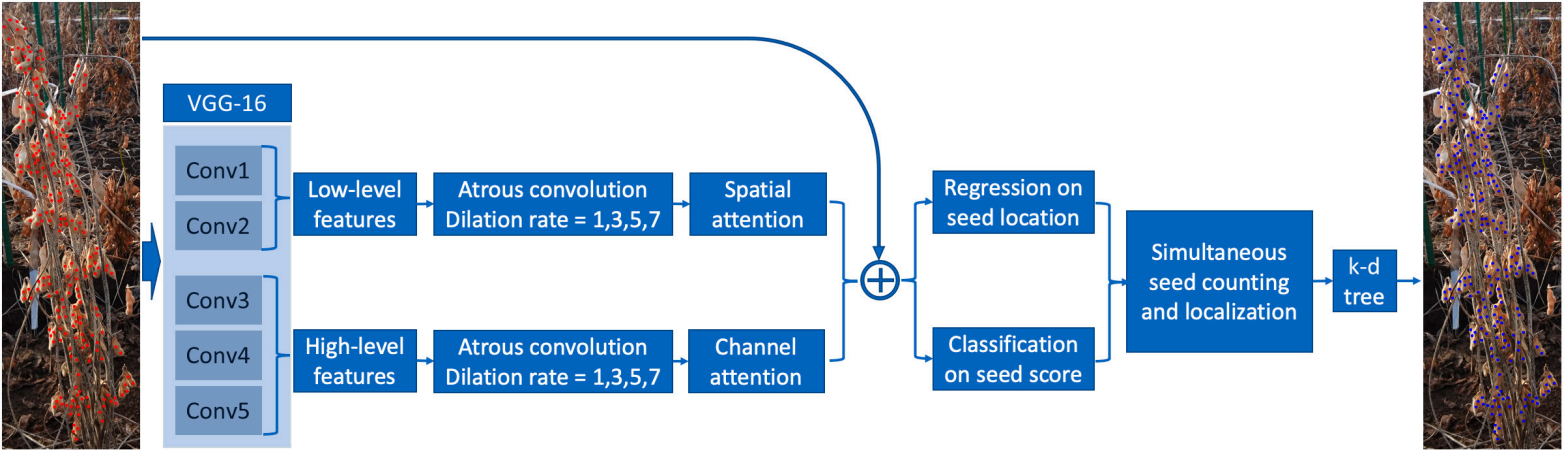
Flowchart of the proposed P2PNet-Soy. The VGG-16 is the base framework for feature extraction. The high- and low-level features were fused first and added to the original image for subsequent localization and counting.

However, full exploration of the advanced concept of P2PNet by directly utilizing it for soybean seed counting is not a straightforward task, especially when the soybean images taken in the field have a complex background, massive disturbance from neighboring plants, different seed sizes, and different degrees of seed overlap, all of which together make the original P2PNet ineffective in actual soybean seed counting. Hence, we proposed the application of several strategies in addition to the original P2PNet to fully adapt it to soybean seed counting, with a special focus on field images (Fig. 3).

Several strategies have been adopted to improve model performance considering the complex situations of field soybean seed counting. First, there are many predicted seed locations stuck around the ground truth. An unsupervised clustering algorithm, the k-d tree [39], was used to find the centers of these closely located predictions to improve final prediction accuracy. Second, only high-level features were used in the original P2PNet for both the regression and the classification tasks. Since higher-level features typically contain global-context-aware information and lower-level features capture more detailed spatial structural information, the combination might fully explore the model potential for soybean seed counting [36]. Third, seed size varied among soybean accessions. Feature extraction methods that consider size differences can also be important for improving model performance. Similar to the approach by Zhao and Wu [36], atrous convolution with dilation rates of 1, 3, 5, and 7 to cover different reception fields on different levels of features was used to obtain the scale-invariant features to deal with seed size variation. Fourth, spatial attention was applied to reduce the noise level of low-level features, and channel attention was adopted to highlight the semantic information of soybean seeds. Spatial and channel attentions were used to effectively prioritize boundary information between target seeds and their background, resulting in better counting performance. Finally, the original image was taken directly as a reference, formatting the entire framework as a residual learning to improve model performance.

### Training and validation of the network

As the images of the soybean plants were taken in the field, cropped individual plant images taken from one side (181 in total) were used for training, and 193 images from the opposite side were used for evaluation. Model performance was evaluated using the mean absolute error (MAE) based on 193 testing images. Finally, model performance in seed counting and localization was visualized in field images obtained directly without cropping.

## Results

**Fig. 4.**
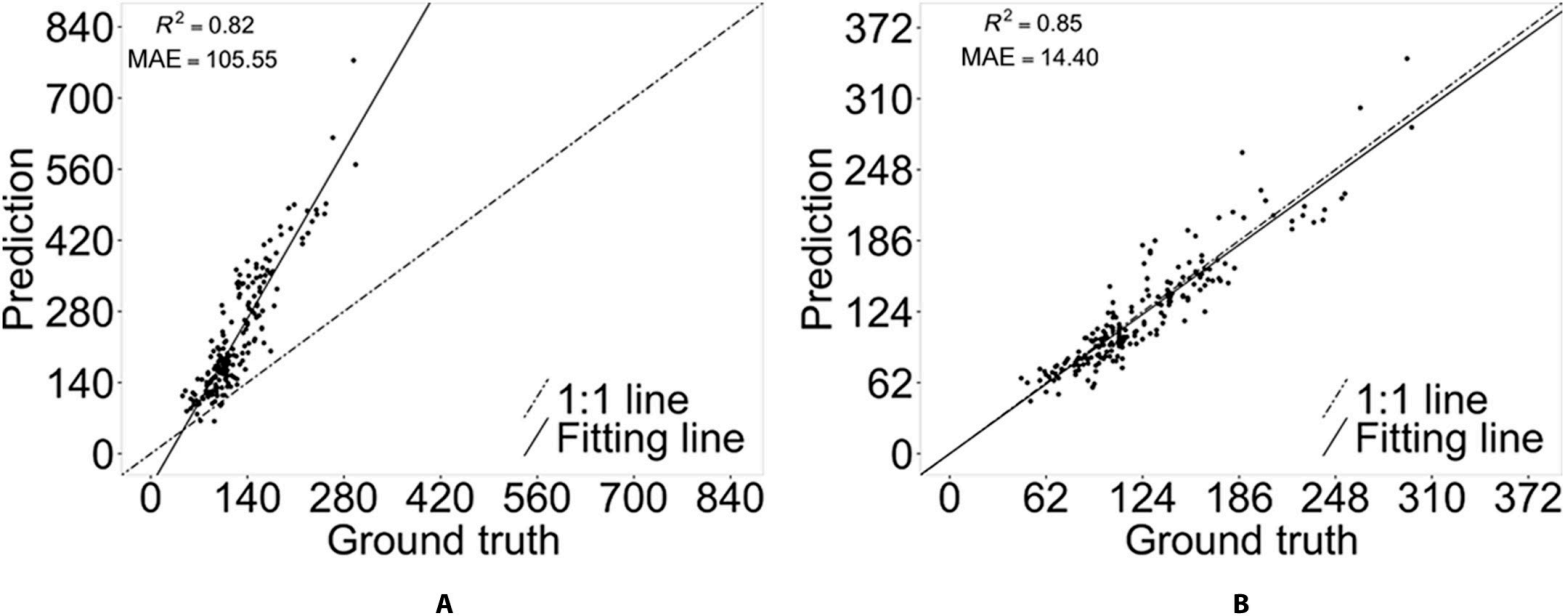
Performance of the original P2PNet. (A) Without k-d tree postprocessing. (B) With k-d tree postprocessing in soybean seed counting on individual plants.

**Fig. 5.**
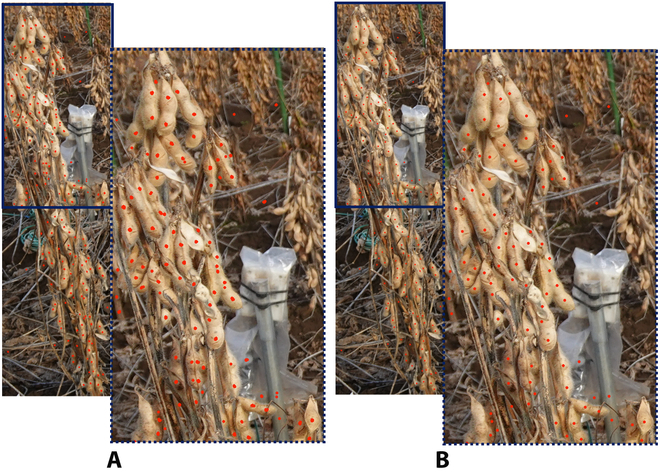
The comparisons between the original P2PNet (A) without postprocessing and (B) with postprocessing in predicting soybean seed number on an individual plant randomly selected from testing dataset. The images in dotted boxes are zoomed-in sections of images inside the solid boxes.

MAE of the original P2PNet was 105.55; however, the correlation between predicted and ground-truth seed counting was still high, with an *R*^2^ of 0.82 (Fig. 4). The major error was due to the inability of the original P2PNet to remove these closely located predictions, as can be seen from the images (Fig. 5A), resulting in a dense “crowd” surrounding the corresponding true locations of these seeds. By applying the k-d tree directly as a postprocessing procedure, the value of MAE decreased sharply to 14.40 and had an improved correlation of 0.85 with their ground-truth annotations (Fig. 4B). However, some seeds that were big in size were missed in these predictions (Fig. 4).

**Fig. 6.**
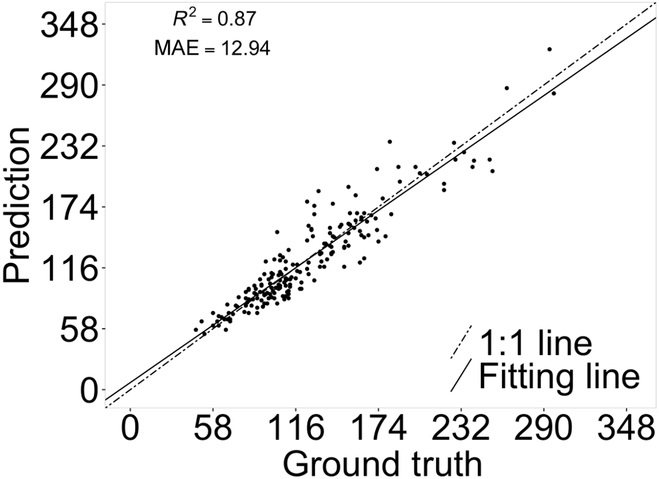
Performance of the P2PNet-Soy in predicting soybean seed number of individual plants.

**Fig. 7.**
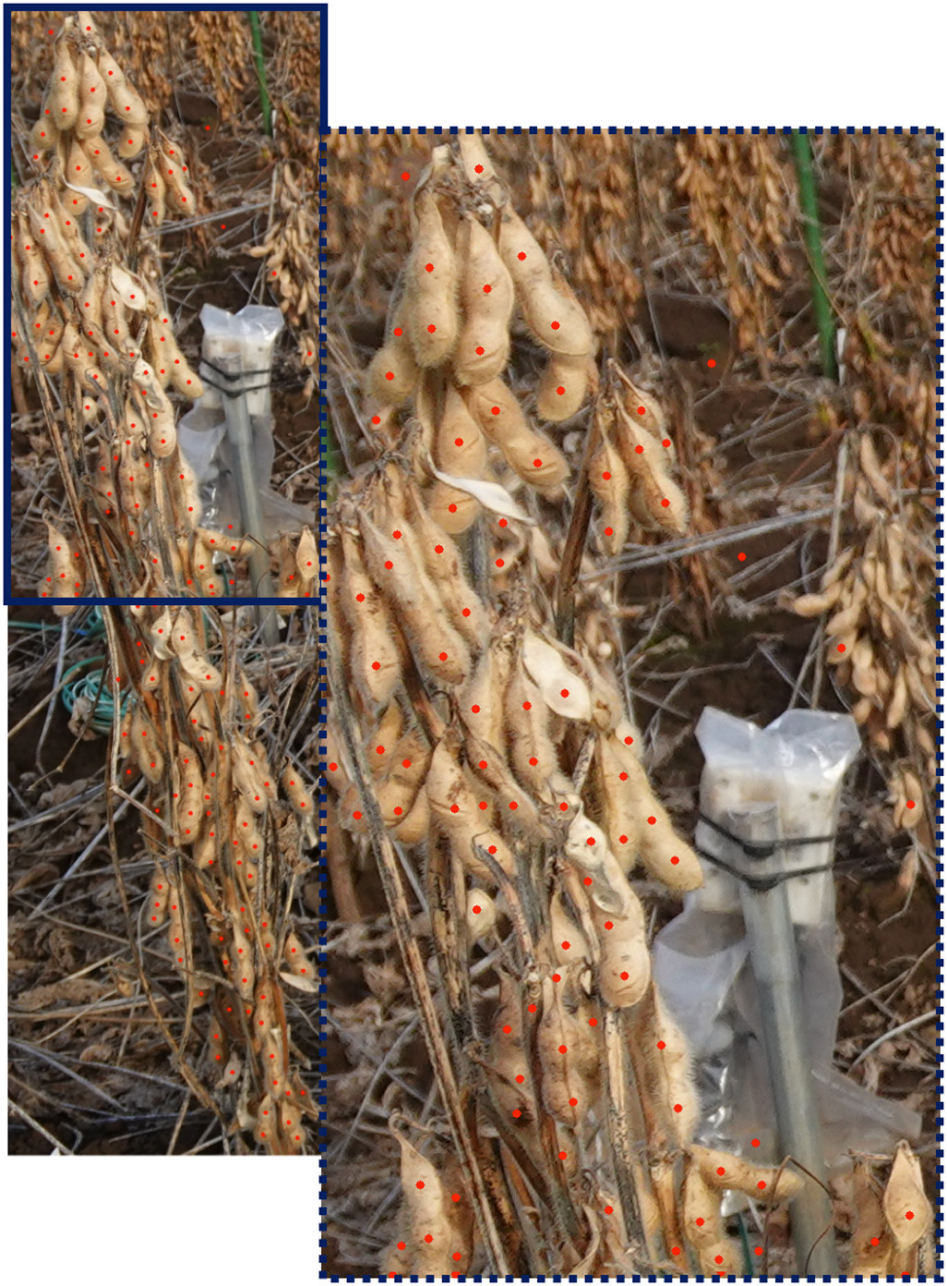
An example of soybean seed prediction by the proposed P2PNet-Soy model. The image in the dotted box on right side is a zoomed-in section of the image inside the solid box of the image on left side.

**Fig. 8.**
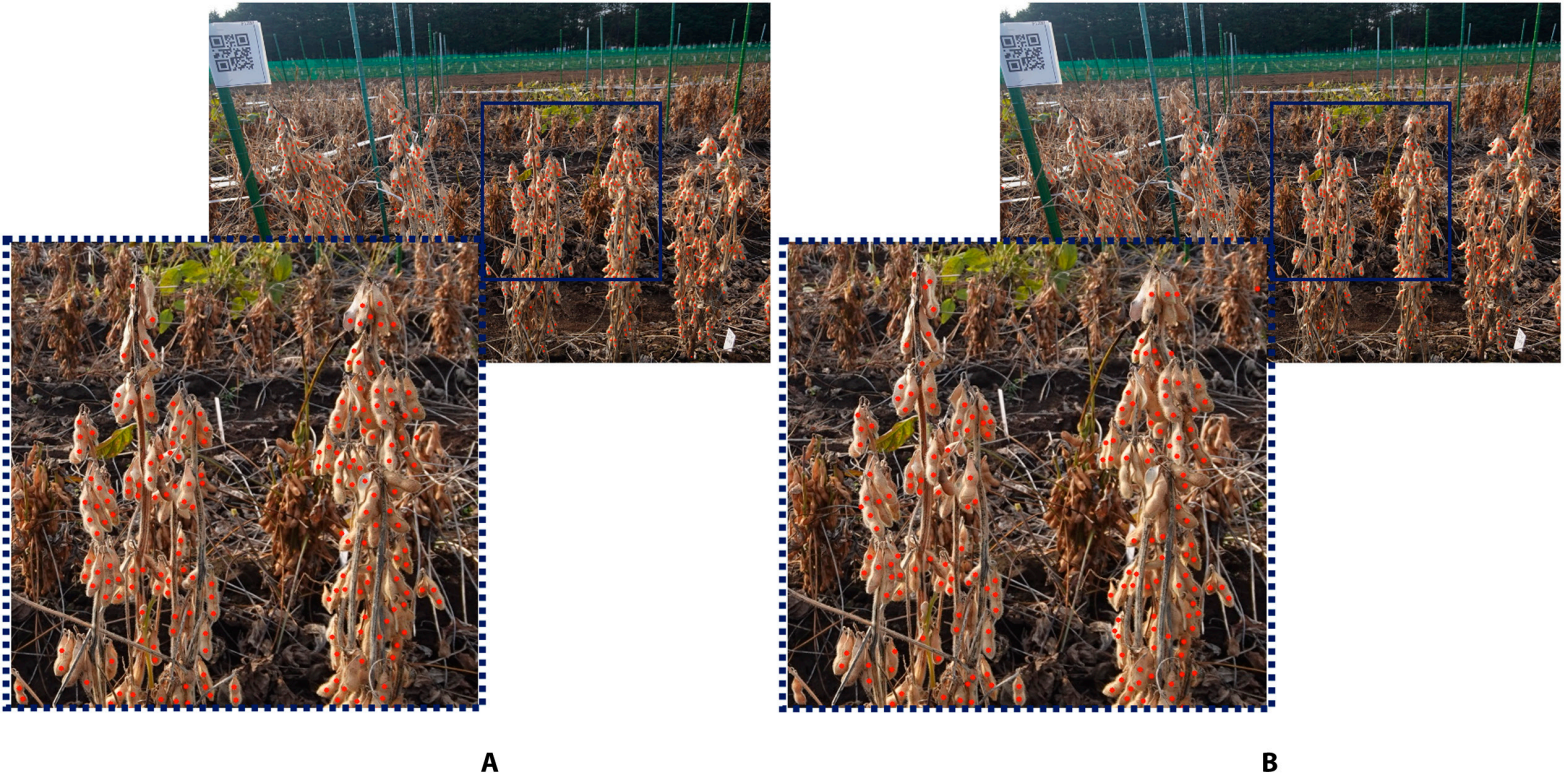
Comparisons of the optimized P2PNet-Soy (A) and the Original P2PNet (B) models with postprocessing on a randomly selected in-field image. The images in dotted boxes are zoomed-in sections of images inside the solid boxes.

The performance of the proposed P2PNet-Soy model was significantly better than that of the original P2PNet, with an MAE value of 12.94 and an *R*^2^ of 0.87; furthermore, the model remained superior even when postprocessing was included in the original P2PNet model (Figs. 6 and 7). Some large seeds that were previously missing in the original P2PNet were detected by the proposed network (Fig. 7). The models also showed very good performance when applied to images taken directly from the field (Fig. 8). The improved performance of the optimized P2PNet-Soy over the original P2PNet model, even with postprocessing, is shown in Fig. 8.

Despite its improved performance, the proposed P2PNet-Soy model was still highly dependent on postprocessing, whereby ablation studies were conducted to further elaborate on the different contributions of each strategy (Table). The MAE value of the original P2PNet was largely reduced by k-d tree postprocessing, and the addition of low-level and multiscale multireception field feature extraction (MSMR) further reduced the value slightly to 14.16 and 14.02, respectively. In contrast, adding spatial attention to low-level features alone increased MAE to 14.53, but it was further reduced to 13.72 when the original image was included directly for soybean seed counting and localization. The addition of channel attention to high-level features further reduced MAE to 12.94, reaching the best performance.

**Table.** Ablation studies on different strategies adopted for improving final model performance.

	Features	Postprocessing	MAE
P2PNet	H	−	105.55
Update1	H	+	14.40
Update 2	H + L	+	14.16
Update 3	H + L + MSMR	+	14.02
Update 4	H + SAL + MSMR	+	14.53
Update 5	H + SAL + MSMR + OI	+	13.72
Update 6	CAH + SAL + MSMR + OI	+	**12.94**

L, low-level features: VGG16-conv1 and VGG16-conv2; H, high-level features: VGG16-conv3, VGG16-conv4, and VGG16-conv5; SAL, spatial attention on low-level features; CAH, channel-wise attention on high-level features; MSMR, multiscale multireception field feature extraction; OI, original image.

## Discussion

Soybean seed counting is important for yield prediction and contributes to more effective farm decision-making. Taking advantage of the latest advancements in crowd counting as the intuitive P2PNet can take the dot-labeled image directly for model training, and soybean seed counting based on infield images was expected to be greatly facilitated. However, such direct transfer led to unsatisfactory performance because the original P2PNet model was not designed for soybean seed counting. Hence, to fully adapt it to soybean seed counting, especially taking advantage of mobile phone cameras to conveniently take photographs in the field, we applied several strategies to upgrade the original P2PNet to P2PNet-Soy, which can be used for field soybean seed counting more effectively and accurately.

The original P2PNet model suffered severely from a large MAE due to the occurrence of overpredictions that were more than 10-fold higher than the corresponding ground-truth values. The major error factor was the low ability of the model to suppress predictions that were far from the corresponding true locations. The k-d tree [39] postprocessing was highly effective in searching for the centers of the overly predicted seeds, thereby reducing the MAE value and localizing the predictions closer to their true locations. Although the *R*^2^ value for the regression between predicted and ground-truth seed counts decreased by 0.2, the accuracy of both predicted seed counts and locations improved significantly. As seed counting and localization are essential for breeding, postprocessing has become indispensable to increase their precision. Further, the Hungarian algorithm [40] was used to assign the predictions to ground-truth seed labels during training; however, it became nonapplicable when the ground-truth seed labels were unavailable for external testing images. The improved performance of the original model after postprocessing confirmed its great convenience for soybean seed counting, especially with dot-annotated labels. As any small upgrading of the model that involves a more effective architecture can be rather difficult, additional postprocessing is not only effective in improving the performance of the model but also, to begin with, a much easier course of action to take.

The more informative extracted features provide a more solid basis for any successful deep-learning framework [35]. The ignorance of different feature levels containing very different information from that in the original input image was another reason explaining the poor performance of the original model. There are 2 separate branches in the original P2PNet, one for classification and the other for regression, and the combination of these 2 branches showed superior experimental performance compared to that of either module by itself [37]. Higher- and lower-level features have different characteristics and are complementary to deep-learning tasks. These low-layer filters detect features such as edges and corners, whereas higher-level filters detect high-level semantic patterns such as parts and objects [41]. Higher-level features are generally more obscure and focus on the general context of the objects; however, they have a fairly low spatial resolution and, consequently, lead to a lower potential to accurately localize large objects or recognize small objects [42]. The addition of lower-level features with detailed texture and location information ultimately promotes model prediction accuracy [36]. The inclusion of different levels of features that fully capture all necessary information satisfying different tasks ensures greater model performance.

Significant improvement in model performance came from the involvement of spatial attention on low-level features and the addition of the original input image. The extracted low-level feature maps contain many details; however, most of them are noises that distract the model from searching for more useful information. Spatial attention was used to help the model focus on edge information and potentially find the boundaries between target seeds and their background for more effective counting [43]. The original image has seldom been captured directly for detection purposes; however, the original images provide more complete spatial information than low-level features [35]. The direct inclusion of the original image reduced the learning load compared with models solely dependent on the extracted features, resulting in the improved performance achieved in this study.

The extraction of multiscale features through atrous convolution [44] to cover the soybean seed size differences is due to either the different accessions in the dataset or the variances within each accession. Atrous convolution with kernels of different size not only increased the receptive field but also ensured that the extracted features were scale-invariant, helping the model to recognize the seed at different scales and improve the robustness of the model against variations in the visible size of the seeds in the image.

Considering the reduction in model MAE value, the postprocessing component showed the highest contribution to the improved performance compared to that of the others. The combination of both higher- and lower-level features and the original input image, together with atrous convolution and attention mechanisms, improved the model performance in soybean seed counting and localization. As suggested by the information contained in the different levels of features extracted by CNN, they should be fused for further improvement of soybean seed counting and localization. We showed the spatial-information-rich lower-level features in improving the model performance. The original image also played an important role in improving model performance. The inclusion of the original image in soybean seed counting and localization highlights its potentially pivotal role in deep learning tasks, as the extracted features are mostly used for different deep learning tasks in lieu of the original image. Postprocessing is unavoidable in many deep learning tasks, either embedded into the main network or added sequentially [45–48]. Wrong predictions that are very close to the corresponding ground-truth values are generally related to network architectures that propose many points in advance, thus leading to the difficulty of their removal during the learning of the network. Further, an advanced architecture should be proposed to reduce the artifacts arising from it, such that additional postprocessing might become unnecessary.

## Conclusion

We have proposed an upgraded P2PNet-Soy method for more effective soybean seed counting and localization with much higher accuracy in comparison to not only the original P2PNet but also other soybean pod counting methods. Infield soybean counting and localization are relatively more difficult than laboratory experiments because of the complex background and overlapping of leaves and other pods. More surprisingly, the trained model also performed excellently on infield images without cropping individual plants out, making it a potential tool for future implementations in the field. However, there are still some limitations of the current method. First, the total count of seeds from the front and back of the plant is certainly an overestimation of the yield and needs to be improved, and the count should be based on the comparison of positional information about seed distribution and other related information. Second, the invisible parts (which never appear in the image) cannot be detected by the algorithm. To solve this problem, we initially considered using videos instead of pictures, because videos can obtain multiple frames from different directions that may make the invisible pod become visible from a certain view angle. This has also been proven in our previous work [49]. Figures 10 and 11 in [49] show how the apple appears and disappears because of the video camera movement. However, for some varieties, the soybean plant structure and position of pods can be very complicated, and any red, green, and blue sensing methods cannot capture all pods. Seeds cannot be detected and counted by the current solution. We may need to consider a more advanced method to solve this problem as a future challenge such as (a) different sensing techniques (technology) such as x-ray or computed tomography to acquire the invisible part of the soybean plant, (b) assistant 3-dimensional modeling to predict the invisible side based on visible information, and (c) a statistical model that can link current detection and counting result from the visible part with real counting number acquired by destructive manual sampling. Third, seed abortion during seed development or seed filling failure can affect yield and easily occurs because of various factors such as environmental stress, disease, and insect injuries. However, it has been difficult to assess its extent because of the time and effort required for the investigation. Our approach also has not been tested for such cases.

We would like to take this research ahead in the future to investigate whether the extent of seed abortion can be quantified easily and whether the physiology and genetics of seed production can be elucidated. Future implementations of the upgraded model for mobile robots should be conducted to decrease human labor involvement in autonomous missions. These effective strategies should also be applied to other counting and localization-related tasks, such as spikelet and tassel counting. Future models that remove postprocessing with simpler architectures should be proposed to facilitate soybean yield prediction by taking advantage of depth cameras or lidar to suppress background information effectively.

## Data Availability

The data used in this study will be available upon request here: https://github.com/UTokyo-FieldPhenomics-Lab/P2PNet-Soy.
